# Is TGF-β1 a Biomarker of Huntington’s Disease Progression?

**DOI:** 10.3390/jcm10133001

**Published:** 2021-07-05

**Authors:** Klaudia Plinta, Andrzej Plewka, Magdalena Wójcik-Pędziwiatr, Nikola Zmarzły, Marcin Rudziński, Monika Rudzińska-Bar

**Affiliations:** 1 Neurology and Stroke Department, Regional Hospital of Saint Hedwig, 45-221 Opole, Poland; klaudia_plinta@o2.pl; 2 Institute of Health Sciences, University of Opole, 45-040 Opole, Poland; aplewka@sum.edu.pl; 3 Department of Neurology, Faculty of Medicine and Health Sciences, Andrzej Frycz Modrzewski Krakow University, 30-705 Krakow, Poland; magdalena.wojcik4@wp.pl; 4 Department of Histology, Cytophysiology and Embryology, Faculty of Medicine, University of Technology in Katowice, 41-800 Zabrze, Poland; nikola.zmarzly@gmail.com; 5 Department of Laryngology, Jagiellonian University Medical College, 30-688 Krakow, Poland; mtrudzin@gmail.com

**Keywords:** Huntington disease, TGF-β1, markers

## Abstract

Huntington’s disease (HD) is an autosomal dominant genetic disease that can be divided into preclinical and symptomatic stages. Due to the diverse HD phenotype, there is an urgent need to identify markers that would independently assess its severity. The aim of this study was to evaluate the use of plasma levels of TGF-β1 in the assessment of HD severity. One hundred HD patients and 40 healthy volunteers were included in the study. All HD patients underwent neurological and cognitive function assessment. TGF-β1 levels were determined in the plasma of all patients. The correlations between TGF-β1 levels and clinical profile and HD severity were also investigated. In symptomatic patients, cognitive decline was demonstrated, while in preclinical patients, no symptoms were found. Plasma levels of TGF-β1 in HD patients did not differ significantly from the control group and did not change with the progression of the disease. In addition, TGF-β1 levels also did not correlate with the severity of motor dysfunction. Positive correlations between plasma TGF-β1 concentration and intensity of cognitive impairment were found, but only in the early disease stage. There was no clear benefit in assessing plasma TGF-β1 levels in HD patients as a marker of disease severity.

## 1. Introduction

Huntington’s disease (HD) is a genetically determined, autosomal dominant neurodegenerative disease. Incidence of HD worldwide is 2.71 per 100,000 people, ranging from 0.4 per 100,000 in Asian populations to 5.7 per 100,000 in North America, Europe and Australia [1]. Lower incidence has been noticed in the Far East countries, i.e., Japan, Taiwan and Hong Kong, with an average incidence of 1–7 patients per million population [1,2].

Primary regions of neurodegeneration in HD patients are the caudate and putamen. The key damage is the extensive and selective atrophy of the striatal GABAergic spinal neurons that regulate the activity of the substantia nigra and globus pallidus [3]. In neuroimaging studies, early involvement of the motor cortex and the occipital lobe was also reported, suggesting widespread brain pathology in HD. In MRI studies of HD patients, decreased striatal volume and white matter changes have been reported even in the preclinical phase of HD [3,4,5].

Motor symptoms of HD include involuntary movements, such as chorea (most common), dystonia, parkinsonism or cerebellar disorders [6,7]. In the initial phase of HD motor manifestation, chorea movements are usually focal, and less often segmental. In the fully symptomatic period, a hypotonic-hyperkinetic picture dominates, which progresses into stiffness with akinesia in later stages of the disease. Diagnosis of HD is based on the clinical picture, supported by a positive family history of chorea and a genetic test that determines the number of CAG repeats. However, it should be remembered that the most common cause of chorea are disturbances during neuroleptic and dopaminergic treatment, and not HD [3].

Assessing severity of the disease and response to treatment using simple and non-invasive methods remains a challenge for researchers studying HD [8,9]. Since the clinical picture of the disease can be very diverse and difficult to determine only by clinical methods, other diagnostic tools such as biomarkers are being explored. One that could be used as a marker on a daily basis to assess disease progression is transforming growth factor β (TGF-β).

About 40 proteins belong to the TGF-β family, including three isoforms of TGF-β [10,11]. TGF-β1 is a cytokine found in the central nervous system, as well as in peripheral tissues. Physiologically, its activity is modulated by age, exercise and disease states. Disturbances in the TGF-β1 level have been noticed in other diseases, such as systemic lupus, scleroderma, bronchial asthma, atherosclerosis, and liver and kidney diseases. In neurology, these disturbances have been studied, among others, in neurodegenerative diseases such as HD, Alzheimer’s disease (AD) or Parkinson’s disease (PD) [10,11,12,13]. Disturbances in the TGF-β1 pathway have been reported in AD associated with extracellular accumulation of fibrillar amyloid β and intracellular deposition of the tau protein. Baseline plasma levels of TGF-β1 in HD patients are lower compared to healthy people. As symptoms develop, plasma concentration of TGF-β1 increases [14,15]. In a study by Chang et al. correlations between plasma TGF-β1 levels and disease duration, age of symptom onset, the number of CAG repeats and the Unified Huntington’s Disease Rating Scale (UHDRS) were also evaluated, however they proved statistically insignificant [16].

The aim of this study was to evaluate the usefulness of plasma levels of TGF-β1 in the assessment of HD severity.

## 2. Materials and Methods

A total of 140 individuals were enrolled in the study, including 100 patients with diagnosed and genetically confirmed HD, treated at the Department of Neurology and Clinic for Neurology of the University Clinical Center of the Medical University of Silesia in Katowice, and 40 healthy volunteers matched for age and sex, with normal neurological examination and no family history of HD or other neurodegenerative disease. Objectives of the study were presented to both groups and written consent was obtained from all study participants. Summary of study group characteristics are presented in Table 1.

### 2.1. Classification and Neurological Examination in HD Patients

In all patients with diagnosed and genetically confirmed HD, neurological examination was performed with the assessment of motor disorders according to the Unified Huntington’s Disease Rating Scale (UHDRS). The following parameters were assessed: pursuit and rapid eye movements (for the left and right eyes respectively), speech disorders, dystonia and chorea divided into the torso, face and limbs, bradykinesia (slowness of movement) and impaired postural reflexes (retropulsion). For each domain, from 0 to 4 points were given (0—no deviations from normal state in the examined parameter, 4—severe disorder). A maximum of 124 points could be obtained in the motor part, and symptomatic phase of HD was diagnosed in those who scored six points or more.

The patients were divided into five groups based on the total score obtained on the motor domain of UHDRS:

1. Preclinical stage—0 points
2. Very early disease stage 1–13 points
3. Early disease stage 14–37 points
4. Intermediate disease stage 38–67 points
5. Advanced disease stage >67 points

In addition, severity of the disease was determined through functional assessment of independence in social and life functions, evaluated on a scale ranging from 10% (a patient requiring help from other people in all life activities) to 100% (an independent patient in domestic and social activities).

### 2.2. Assessment of Cognitive Functions and Mood Disorders in HD Patients

The following tools were used to assess cognitive functions in HD patients:

Screening tests:

1. Mini-Mental State Examination (MMSE)
2. Montreal Cognitive Assessment (MoCA)
3. Clock Drawing Test (CDT)

Detailed tests:

1. Symbol Digital Modality Test (SDMT), assessing visual-spatial attention based on matching digits to a sequence of symbols in 90 s as in a formula consisting of 9 characters combined with digits
2. Verbal fluency tests determining verbal fluency (VF) and phonemic fluency (Total Fluency, TF)
3. Trail Making Test (TMT) part one (connecting points numbered from 1 to 25 in sequential order) and two (connecting points in order while alternating between numbers and letters, i.e., 1-A-2-B-3-C-etc.)
4. Stroop test (ST) parts one, two and three. In the first part, a sheet of paper is given to the examined person, which contains squares filled with colored ink. The task is to recognize as many consecutive colors as possible within 45 s. In the second part, the examined person receives a sheet with one hundred words, divided into ten lines with the names of the colors—the task is to read as many words as possible within 45 s. In the last part of the test, there are words that represent colors written in a different ink color than the word they actually mean—the task is to recognize the ink color with which the word is written.

### 2.3. Biochemical Assessment

Plasma levels of TGF-β1 were determined using professional ELISA kits, according to the manufacturer’s protocol (Diaclone SAS, Besancon Cedex, France; cat. no. 650.010.096).

### 2.4. Statistical Analysis

Statistical analysis was performed using Statistica by StatSoft, version 13.1. The Kolmogorov–Smirnov test was used to determine the data distribution of the studied groups. In case of normal data distribution and homogeneity of variance, Student’s *t*-test (for two groups) and ANOVA, and, if necessary, post-hoc comparisons with NIR test (for three groups), were performed. In case of deviations from normal distribution, the Mann–Whitney U test or Kruskal–Wallis test with Dunn’s post-hoc analysis were used for two and three groups, respectively. Correlation strength was evaluated with Pearson’s test for normal distribution and Spearman’s test otherwise. Correlations with *p*-value less than or equal to 0.05 were considered statistically significant.

## 3. Results

### 3.1. Basic Results of the Patients

The following subgroups were distinguished among the study group based on the UHDRS: preclinical stage, 12 patients; very early disease stage, 12 patients; early disease stage, 23 patients; intermediate disease stage, 33 patients; and advanced disease stage, 20 patients.

The average number of repeats of the larger allele was 44, while the average score obtained on the UHDRS motor scale was 40.39. The mean disease duration was 9.53 years. Collective results of the study group are presented in Table 1.

### 3.2. Neurological Examination of HD Patients

The results analysis of movement disorders assessed by motor UHDRS showed an increase in neurological deficit along with the severity of HD. In patients in the very early disease stage, no significant oromandibular movement disorders were found. Most patients at this stage did not experience significant chorea of the body (with the exception of chorea of the face, mainly squeezing the eyelids and raising the eyebrows, and of the upper limbs, mainly distal parts), gait disturbances or postural reflexes. In most patients, no increase in muscle tone, and no significant slowing down of movement or dystonia (typical of higher stages of HD) was noted.

In patients in the early disease stage, intensification of eye movement disorders was observed. In this group of patients, within the movement disorders, chorea (mainly of the limbs) predominated over dystonia. Similarly, greater deficits were found in tandem walking than in normal walking, and impaired alternating movements and the efficiency of the Luria’s test were also evident.

In the intermediate group, there was a further deterioration in eye movements, still more evident in saccadic movements than in pursuit movements. In addition, disturbances in tongue protrusion or alternating movements of the upper limbs were intensified. Dystonic disorders and increased chorea movements, and hence gait disturbances and postural reflexes, were evident.

In patients with advanced disease, increased eye movement disorders were observed. In addition, further impairment of alternating movements was observed, as well as an increase in dystonic and choreic disorders and muscle tone. Gait, tandem gait and postural reflexes were significantly impaired. Table 2 presents the results of neurological assessment according to the motor UHDRS. Results of patients from the preclinical group were not presented as there were no abnormalities in the neurological examination in this group.

### 3.3. Assessment of Cognitive Functions in HD Patients

In the average assessment of all the patients, they scored 23.75 ± 5.92 points on the MMSE scale, i.e., the result on the borderline of mild cognitive impairment (MCI) and mild dementia. In preclinical patients, no significant abnormalities in the MMSE test were found. Only in the intermediate group, the deterioration of cognitive functions, suggesting MCI, was noted. In the case of the MoCA assessment, the disorders were already visible in the very early stage of HD. CDT results showed no deviations in the initial stages of the disease. The result suggesting incorrect execution of the CDT was noticed only in the intermediate group. Assuming a correct SDMT result of a minimum of 47, the mean score of all HD patients was more than 50% lower. When analyzing individual stages of the disease, no disturbances in SDMT were found in patients in the preclinical stage. In patients in the very early disease stage, the mean score was lower than the expected norm.

In subsequent stages of the disease, the number of correct answers decreased twofold, whereas during the transition from intermediate to advanced stage, an even greater severity of the deficit was observed. The fewest errors were made by patients in the advanced disease stage, which results from a much smaller number of all matches in this group of patients, i.e., the average number of correct matches was less than 2 points, and incorrect matches was 0.15. For comparison, in the very early disease stage, on average about 44 correct matches were given, with the number of incorrect matches being 0.9. Patients in the advanced stage showed less global matches (extended latency of the task execution), and thus had fewer opportunities for error—this phenomenon was also visible in further tests. Additionally, attention distraction was frequently observed in patients in the initial symptomatic stage of HD.

Analysis of Stroop test results revealed that there was no significant increase in the number of incorrect answers between different disease stages; however, there was a reduction in the number of correct answers. In the Stroop color naming test, the average score for the number of correct responses in all patients was approximately half of that in preclinical patients. In the group of preclinical patients, no incorrect responses were found. During the transition of patients to the advanced disease stage, a large reduction in the number of correct responses was noted.

In the Stroop word test, results were similar to the color test, however, patients scored more points, which is consistent with the general rule of the test—reading words is easier than recognizing colors. Errors in the word test were not reported until the early stage of HD. The least correct answers were recorded in the third part of the Stroop test—interference, which is also consistent with the test assumptions. Again, a large reduction in correct responses was observed during the transition from the intermediate to advanced stage, and incorrect responses were already noted in the early stage of the disease. Results of these tests are summarized in Table 3.

### 3.4. Plasma Levels of TGF-β1 in HD Patients vs. Control Group

#### 3.4.1. Basic Results

**Figure 1:**
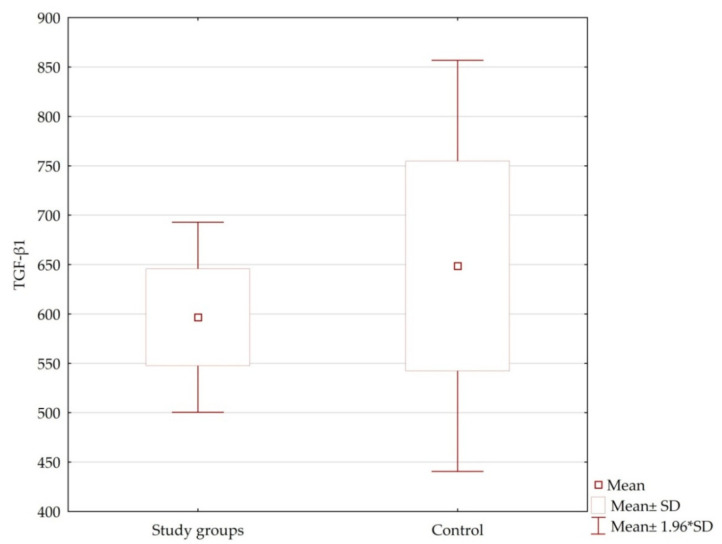
Plasma levels of TGF-β1 in the study groups and control.

**Figure 2:**
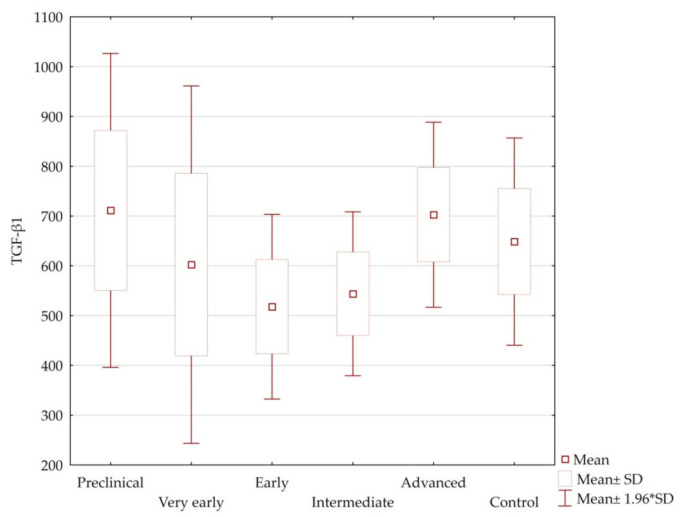
Plasma levels of TGF-β1 in the individual HD stages and control group.

Plasma levels of TGF-β1 in patients were not dependent on age, sex, number of CAG repeats of the large allele, age of symptom onset, or HD stage (*p* = n.s.) (Table 4, Figure 1 and Figure 2). Among healthy volunteers, there was no statistically significant relationship between plasma levels of TGF-β1 and age or gender. Highest levels of this cytokine were recorded in the preclinical and advanced groups. Lowest TGF-β1 levels were noted in patients in the early disease stage. In preclinical and very early disease groups, the range of TGF-β1 level was much wider than that of the other groups of patients.

#### 3.4.2. Correlations between Plasma Levels of TGF-β1 and the Results of Neurological Examination and Cognitive Function Tests

Correlations between TGF-β1 levels and performed tests were not analyzed in the group of preclinical patients (motor UHDRS = 0). The greatest number of correlations was recorded in the advanced and early stages of HD. Correlations between plasma levels of TGF-β1 and each parameter of the motor UHDRS as well as cognitive tests are summarized in Table 5, Table 6 and Table 7. Only statistically significant correlations are presented below. In early HD, higher levels of TGF-β1 correlated with greater intensity of some motor symptoms (e.g., tapping), while in advanced HD the opposite relationship was observed (higher levels of TGF-β1 correlated with lower intensity of some motor symptoms, e.g., dystonia and tapping). In patients with early disease, plasma levels of TGF-β1 correlated positively with the severity of abnormalities in the pronation/supination test (*p* = 0.02, R = 0.46; Table 5) and slowing down of the upper limb (*p* = 0.01, R = 0.48), i.e., higher levels were observed in patients with more severe motor symptoms.

In the intermediate group, higher plasma levels of TGF-β1 were observed in patients with more severe lower limb dystonia (*p* = 0.03, R = 0.36). Similarly, positive correlation was found between plasma levels of TGF-β1 and the intensity of tongue protrusion disorders (*p* = 0.01, R = 0.53) in the advanced HD stage. However, other motor symptoms in the advanced stage showed negative correlations with plasma levels of TGF-β1, and higher levels were associated with lower tapping intensity of both upper limbs (*p* = 0.03, R = −0.47) and less severe dystonia of the trunk (*p* = 0.04, R = 0.45) and lower limb (*p* = 0.04, R = 0.44).

In the analysis of the relationship between plasma levels of TGF-β1 and elements of neuropsychological assessment, increased levels of TGF-β1 in the early stage of HD positively correlated with the results of MMSE (*p* = 0.03, R = 0.52) and CDT (*p* = 0.01, R = 0.56). Correlations were also observed between levels of TGF-β1 and independence scale (*p* < 0.01, R = 0.45) and the Beck scale (*p* = 0.01, R = −0.66) in the intermediate and advanced stages, respectively (Table 6).

In the analysis of the association between plasma levels of TGF-β1 and cognitive functions, the former correlated negatively with the number of correct answers in the Stroop word reading test and positively with the number of errors in the trail making test in patients in the early disease stage (Table 7). High levels of TGF-β1 in the early disease stage were more common in patients who were less likely to give correct answers in the Stroop word reading test (*p* = 0.04, R = −0.43) and were more likely to make mistakes in the trail making test (*p* = 0.04, R = 0.42). In the advanced stage, higher plasma levels of TGF-β1 occurred in patients who were less likely to self-correct wrong responses in the Stroop test (*p* = 0.04, R = −0.46).

The only correlations indicating the neuroprotective effect of high TGF-β1 concentration were found in verbal fluency tests. Increased plasma levels of TGF-β1 inversely correlated with the number of incorrect repetitions in the verbal fluency test, both in the early disease stage (*p* = 0.04, R = −0.43) and in the advanced stage (*p* = 0.01, R = −0.57). In the intermediate group, the above correlation was positive (*p* = 0.02, R = 0.39). Higher levels of TGF-β1 were more common in people who less frequently revealed perseveration, i.e., pathological repetitions indicating a worse cognitive state of the patient.

#### 3.4.3. Results Summary

In patients in the initial clinical stage of HD, movement disorders (chorea), mainly of the face and distal parts of the limbs, were the first to be reported. Eye movement (predominantly saccadic) and tandem gait disorders were already visible in the initial stage of the symptomatic phase of the disease. In the advanced phase of HD, slowness, dystonic disorders, and gait impairment predominate.

In the screening of cognitive functions in the early stage of HD, patients obtained worse MoCA scores than MMSE, demonstrating the greater usefulness of the Montreal scale in assessing early cognitive impairment in HD. The clock drawing test showed no deviations in the initial stages of the disease. In a detailed assessment of cognitive functions (SDMT, VF, TF, TMT, Stroop tests), the first deviations were noted in the very early or early stage of HD, but without significant disturbances in the preclinical stage.

The study showed no statistically significant differences in plasma levels of TGF-β1 between HD patients and the control group, or between individual stages of HD. In addition, no significant correlations have been found between levels of TGF-β1 and the severity of symptoms assessed according to the motor UHDRS. Only correlations between plasma levels of TGF-β1 and individual motor symptoms at different stages of HD were revealed. Similarly, correlations occurred between TGF-β1 level and the assessment of cognitive functions in screening and detailed tests.

In HD patients, coexistence of higher plasma levels of TGF-β1 with better neurological and cognitive performance in the early stages of the disease was reported. This may suggest a degree of neuroprotective effect of TGF-β1, which is exhausted in the later stages of the disease.

## 4. Discussion

Transforming growth factor β is a pleiotropic cytokine that exhibits a very wide range of functions in both physiological and pathological processes. It participates in the regulation of proliferation, apoptosis, and differentiation, as well as adhesion, which is possible due to canonical signaling with SMAD proteins and non-canonical signaling via phosphoinositide 3-kinase (PI3K), protein kinase B (PKB) and mitogen-activated protein kinases (MAPKs) [17]. Many studies currently focus on the participation of TGF-β1 in the development of cancer [18], however, an important aspect is also the possible neuroprotective effect of this cytokine [19]. The determination of TGF-β1 level change may be useful in assessing HD severity and will help to better understand the mechanisms involved in the development of HD.

In this study, we analyzed plasma levels of TGF-β1 in patients with diagnosed and genetically confirmed HD. The obtained results were analyzed against the neurological assessment on the motor UHDRS and its other subscales, as well as selected elements of the neuropsychological assessment—cognitive functions. There were no statistically significant differences in plasma levels of TGF-β1 between HD patients and the control group, or between individual stages of the disease, which is not surprising as similar results were also obtained in other laboratories [8,9,14,15,16,20,21,22,23].

In the study by Chang et al., transition into the clinical phase of HD was associated with an increase in plasma levels of TGF-β1, and its global level in HD patients was higher compared to control group. Similarly, levels of TGF-β1 in the preclinical stage were also higher than in the non-HD individuals. The study, however, does not analyze the distribution of plasma levels of TGF-β1 across different disease stages in symptomatic patients. Plasma levels of TGF-β1 also did not correlate with the duration of the disease, age of the symptom onset and the UHDRS scales [16].

On the other hand, in the study by Battaglia et al., serum levels of TGF-β1 in HD patients were lower than in the control group, similar to our study. Battaglia et al. reported the lowest levels of TGF-β1 in preclinical patients, while an increase was noted during transition to the symptomatic phase, which is contrary to our results. In the same study, levels of TGF-β1 in fully symptomatic patients were comparable to the control group. TGF-β1 level in the preclinical stage was significantly different compared to the other advancement groups [14]. This has not been demonstrated in our study, where no statistically significant differences were found between any disease stage and the control group.

In our study, the relationship between plasma levels of TGF-β1 and the neurological assessment and cognitive functions was assessed. The obtained data did not show any correlations between the TGF-β1 level and the number of CAG repeats, disease duration and the age of symptom onset in all patients or at different stages of HD. In the case of motor disorders, the highest number of correlations was noted in the group of advanced patients. A correlation between higher TGF-β1 level and greater intensity of tongue protrusion disorders was observed. However, increased levels of TGF-β1 in advanced patients were associated with lower severity of dystonic disorders of the trunk and lower limb, as well as a higher tapping speed of the fingers of the upper limbs. In the group of early HD stage, an inverse correlation was observed. Greater intensity of tapping disorders and upper limb pronation/supination test scores were associated with higher plasma levels of TGF-β1, i.e., higher concentrations coexisted with more severe motor symptoms. Similarly, in the intermediate group, high levels of TGF-β1 were associated with greater severity of lower limb dystonia. This may suggest that TGF-β1 concentration depends on the disease stage. In the preclinical and very early stages, TGF-β1 level is high (similar to the control group) and then gradually decreases with the severity of symptoms; hence the hypothesis that in the first stages of HD (preclinical and very early), TGF-β1 has a neuroprotective effect that gradually diminishes in the symptomatic stages of the disease as plasma levels of TGF-β1 decrease. The loss of neuroprotective properties may be related to the lowest concentration of TGF-β1 in these stages, as well as probable independent loss of the neuroprotective functions of this cytokine.

It is worth emphasizing that ours is one of the few studies assessing correlations between plasma levels of TGF-β1 and each element of the neurological test in the motor UHDRS. Despite showing a small number of these correlations, described abnormalities may be important for monitoring the entire course of the disease. Tapping, dystonic and tongue protrusion disorders in HD patients have been studied before. In a study by Reilman et al. from 2010, the strength and duration of tongue protrusion in HD patients was instrumentally evaluated, demonstrating the diagnostic value of the assessment in fully symptomatic (classification of the severity of movement disorders) and preclinical (verification of carrier status in people at risk of HD) patients [24]. A similar diagnostic value was demonstrated for rapid alternating movements of the upper limbs. A study by Bechtel et al. described a relationship between severity of tapping disorders and bilateral atrophy of the caudate nucleus and putamen within the gray matter, a reduction in the volume of the internal and external capsule, as well as atrophy of the superior frontal and parietal postcentral gyrus, visualized in imaging studies in HD patients [25]. In turn, dystonic disorders are the second most common (after chorea) motor disorder in HD, which also require an independent monitoring tool. In our study, we showed a relationship between plasma levels of TGF-β1 and described movement disorders, which may be helpful in the search for a marker of movement disorder severity. It may be also used as a tool for monitoring the response to the proposed treatment of symptoms, and possibly for determining the causes in the future [26]. However, the factor limiting use of this cytokine is certainly the lack of a statistically significant correlation in other disease stages.

Progressive neurodegeneration in the brain of preclinical patients leads to a decrease in TGF-β1 levels. This is accompanied by the exhaustion of compensatory possibilities and transition to the symptomatic stage of the disease, which may be related to the blocking of TGF-β1 expression in the CNS [10]. Correlations between plasma levels of TGF-β1 and the results of cognitive function tests reported in our study may suggest its protective effect in the early stages of the disease, where an increase in this cytokine would correspond with better performance. However, in the advanced disease stage, this protection may be destabilized, which would be indicated by negative correlations between the plasma level of TGF-β1 and parameters assessing cognitive functions. During this period of the disease, levels of TGF-β1 were higher in patients with lower MMSE scores. This may be related to the unblocking of the production or secretion of TGF-β1 when neurons are already affected by progressive damage. Due to the extent of neurodegeneration, it is not possible to compensate for this process by the action of TGF-β1 [10].

The neuroprotective effect in HD was also demonstrated in a study by Naphade et al. One of the causes of CNS pathology is the neurotoxicity of breakdown products of mutant huntingtin, mainly in the striatum and cerebral cortex. Matrix metalloproteinases (MMPs) are responsible for the degradation of huntingtin. Their activity in HD is disrupted, mainly through level changes in tissue MMP inhibitors (TIMPs). It was shown that exogenous TGF-β1 caused an increase in TIMPs, which may explain the neuroprotective effect of higher concentrations of TGF-β1 in CNS [27]. On the other hand, Wachs et al. showed that increased levels of TGF-β1 in the brain of HD animal models suppressed proliferation of progenitor cells, thus enhancing the process of neurodegeneration and inhibiting the repair capacity of the brain. In the preclinical stage of the disease, this is compensated by the increased proliferation of neuroblasts [28]. This thesis would be consistent with the results obtained in our study, where in preclinical patients the levels of TGF-β1 were higher compared to patients in the early disease stage, and the levels gradually increased with the clinical symptoms of HD. This may be at least partially related to increased TGF-β1 signaling within progenitor cells [29].

A limitation in our study may be the effect of drugs or inflammation on the plasma levels of cytokines. In the assessment of biochemical parameters, including growth factors, it is important to analyze the medications taken by patients due to the possible iatrogenic effect on the level of the assessed factors. To date, there is no causal treatment for HD; only symptomatic treatment is available. The most commonly used drugs include tiapride, tetrabenazine and neuroleptics—mainly of the new generation, and a representative of classic neuroleptics—haloperidol. However, the study by Tourjman et al. analyzed the effect of dopamine antagonists on the level of biochemical parameters in schizophrenia and no effect of antidopaminergic agents on the levels of TGF-β1 in the blood plasma was noted [30]. However, it is uncertain whether a similar relationship with the lack of anti-dopaminergic drug effects exists in HD. Another important factor that could modify the level of blood cytokines are pro-inflammatory states, such as infection or autoimmune diseases. By specifying the inclusion and exclusion criteria for the study, attempts were made to minimize the impact of other disease processes on the cytokine level result; however, some patients may not admit or know about the ongoing inflammatory process.

## 5. Conclusions

Plasma levels of TGF-β1 in HD patients did not differ significantly from the control group and did not change significantly with the progression of the disease. This was not consistent with findings presented by other authors who demonstrated a decrease in TGF-β1 concentration in HD patients.

Plasma levels of TGF-β1 in HD patients did not correlate with the severity of motor dysfunction evaluated using the motor part of UHDRS. Positive correlation between plasma TGF-β1 concentration and intensity of cognitive impairment was found on MMSE and CDT, but this was only observed in the early disease stage.

## Figures and Tables

**Table 1:** Characteristics of the study group divided into HD stages.

Variables/ HD Stages	All HD		Preclinical		Very Early		Early		Intermediate		Advanced	
	$\bar{X}$ ± SD	Range	$\bar{X}$ ± SD	Range	$\bar{X}$ ± SD	Range	$\bar{X}$ ± SD	Range	$\bar{X}$ ± SD	Range	$\bar{X}$ ± SD	Range
Age	46.87 ± 13.70	20–73	40.33 ± 1.52	19–42	32.83 ± 10.93	20–55	54.84 ± 9.73	33–70	47.28 ± 12.75	22–66	54.20 ± 16.33	35–69
Age of symptom onset	40.05 ± 18.54	19–68	-	-	30.33 ± 1.07	20–50	49.00 ± 9.26	33–65	35.33 ± 11.20	30–52	40.25 ± 3.36	39–60
Disease duration	9.53 ± 6.71	0–25	-	-	6.60 ± 8.26	1–25	6.16 ± 4.26	1–19	9.75 ± 6.22	2–26	11.85 ± 5.35	4–21
CAG repeats small allele	18.03 ± 2.84	12–30	17.50 ± 0.83	13–27	17.77 ± 3.07	12–23	18.36 ± 4.16	14–30	18.25 ± 3.36	15–23	17.26 ± 1.90	15–22
CAG repeats large allele	44.40 ± 4.28	40–63	41.83 ± 1.16	40–60	45.11 ± 3.95	40–52	43.21 ± 3.10	40–50	45.12 ± 1.01	40–63	45.53 ± 4.27	41–54
Motor UHDRS	40.39 ± 28.84	0–102	-	-	4.66 ± 2.70	1–9	26.38 ± 6.03	14–35	52.95 ± 7.92	39–67	76.80 ± 2.77	74–80
Years of study	13.51 ± 3.21	8–27	18.00 ± 3.68	13–27	15.00 ± 1.78	12–18	14.23 ± 2.68	11–17	13.00 ± 2.79	8–19	12.40 ± 3.36	8–17
MMSE	23.75 ± 5.92	0–30	29.87 ± 0.35	29–30	28.22 ± 1.78	25–30	27.11 ± 2.26	22–30	22.62 ± 4.52	7–29	14.58 ± 4.62	8–22
MoCA	23.98 ± 6.15	0–30	29.00 ± 1.78	25–30	26.71 ± 4.64	17–30	26.92 ± 2.55	20–30	21.80 ± 5.22	7–30	15.00 ± 6.32	9–28
Beck’s scale	5.43 ± 6.09	0–63	3.54 ± 4.43	0–12	6.54 ± 8.04	0–26	3.09 ± 4.01	0–12	5.70 ± 5.38	0–18	5.90 ± 6.47	0–16
Clock drawing test	8.1 ± 2.66	0–10	10 ± 0	10–10	9.63 ± 1.20	6–10	9.15 ± 1.77	5–10	6.40 ± 6.34	3–10	4.18 ± 2.71	0–8
Functional assessment	17.95 ± 6.21	4–25	25 ± 0	25	24.75 ± 9.33	22–25	21.61 ± 3.47	15–25	16.28 ± 5.94	8–25	11.20 ± 5.49	4–19
Independence scale	74.76 ± 16.22	45–100	100 ± 0	100	90.00 ± 6.35	89–100	86.15 ± 9.38	70–100	67.61 ± 13.65	45–90	60.00 ± 7.90	50–70

$\bar{X}$ ± SD—mean ± standard deviation, UHDRS—Unified Huntington Disease Rating Scale, MMSE—Mini-Mental State Examination, MoCA—Montreal Cognitive Assessment.

**Table 2:** The results of the motor UHDRS assessment in HD patients.

Variables/HD Stages	All HD	Very Early	Early	Intermediate	Advanced
	$\bar{X}$ ± SD				
Vertical pursuit	1.13 ± 1.06	0.25 ± 0.45	0.56 ± 0.58	1.60 ± 1.24	2.20 ± 1.00
Horizontal pursuit	1.14 ± 1.09	0.16 ± 0.38	0.65 ± 0.57	1.60 ± 0.50	2.20 ± 1.00
Vertical saccades initiation	1.70 ± 1.29	0.25 ± 0.45	1.04 ± 0.47	2.36 ± 2.80	3.25 ± 0.55
Horizontal saccades initiation	1.67 ± 1.25	0.33 ± 0.65	1.04 ± 0.47	2.27 ± 1.14	3.20 ± 0.61
Speed of horizontal saccades	1.54 ± 1.18	0.25 ± 0.45	1.04 ± 0.47	2.09 ± 2.39	2.90 ± 0.78
Speed of vertical saccades	1.58 ± 1.2	0.25 ± 0.45	1.08 ± 0.51	2.12 ± 0.25	3.00 ± 0.72
Dysarthria	1.20 ± 1.02	0.16 ± 0.38	0.82 ± 0.49	1.45 ± 0.82	2.55 ± 0.82
Tongue protrusion	1.40 ± 1.36	0 ± 0	0.52 ± 0.84	2.00 ± 0.93	3.10 ± 0.55
Bradykinesia (Tapping) of the right upper limb	1.78 ± 1.42	0.25 ± 0.45	1.13 ± 0.54	2.18 ± 0.82	3.85 ± 0.36
Bradykinesia (Tapping) of the left upper limb	1.91 ± 1.48	0.25 ± 0.62	1.30 ± 0.82	2.45 ± 0.71	3.85 ± 0.36
Pronation/supination of the left upper limb	1.72 ± 1.29	0.33 ± 0.49	1.26 ± 0.61	2.09 ± 0.76	3.50 ± 0.68
Pronation/supination of the right upper limb	1.87 ± 1.33	0.33 ± 0.49	1.43 ± 1.33	2.39 ± 0.78	3.55 ± 0.68
Luria’s test	1.87 ± 1.64	0.50 ± 0.90	1.60 ± 1.64	2.12 ± 0.61	3.70 ± 0.57
Left upper limb stiffness	0.93 ± 1.06	0.08 ± 0.28	0.60 ± 1.06	1.06 ± 0.93	2.15 ± 1.08
Right upper limb stiffness	0.96 ± 1.05	0.08 ± 0.28	0.60 ± 1.05	1.15 ± 0.84	2.15 ± 1.08
Bradykinesia	1.44 ± 1.19	0.41 ± 0.66	1.21 ± 1.19	1.66 ± 0.90	2.80 ± 0.83
Torso dystonia	0.49 ± 0.78	0.08 ± 0.28	0.26 ± 0.78	0.54 ± 0.67	1.20 ± 0.95
Right upper limb dystonia	0.64 ± 1.03	0 ± 0	0.17 ± 1.03	0.63 ± 0.60	1.95 ± 1.27
Left upper limb dystonia	0.69 ± 1.09	0 ± 0	0.13 ± 1.09	0.75 ± 0.92	2.05 ± 1.27
Right lower limb dystonia	0.61 ± 0.99	0 ± 0	0.17 ± 0.99	0.69 ± 0.89	1.70 ± 1.21
Left lower limb dystonia	0.65 ± 1.02	0 ± 0	0.30 ± 1.02	0.72 ± 0.87	1.70 ± 1.17
Facial chorea	1.21 ± 1.09	0.08 ± 0.28	1.17 ± 1.09	1.51 ± 0.95	2.15 ± 1.13
Oral-buccal-lingual chorea	1.29 ± 1.24	0 ± 0	1.17 ± 1.24	1.57 ± 0.71	2.50 ± 1.27
Torso chorea	1.30 ± 1.13	0 ± 0	1.17 ± 1.13	1.81 ± 0.82	2.15 ± 1.26
Left upper limb chorea	1.44 ± 1.53	0.16 ± 0.38	1.56 ± 1.53	2.00 ± 0.93	2.00 ± 1.52
Right upper limb chorea	1.27 ± 1.17	0.08 ± 0.28	1.04 ± 1.17	1.87 ± 0.95	2.00 ± 1.52
Left lower limb chorea	1.38 ± 1.19	0 ± 0	1.26 ± 1.19	1.90 ± 1.06	2.30 ± 1.30
Right lower limb chorea	1.37 ± 1.17	0 ± 0	1.21 ± 1.17	1.87 ± 1.00	2.35 ± 1.30
Gait	1.27 ± 1.04	0.08 ± 0.28	0.82 ± 1.04	1.69 ± 0.96	2.55 ± 0.68
Tandem gait	1.58 ± 1.27	0.25 ± 0.45	1.04 ± 1.27	1.96 ± 0.76	3.30 ± 0.57
Retropulsion	1.11 ± 1.05	0 ± 0	0.86 ± 1.05	1.24 ± 0.86	2.50 ± 0.88

$\bar{X}$ ± SD—mean ± standard deviation.

**Table 3:** Results of neuropsychological assessment depending on the HD stage.

Variables/HD Stages	All HD	Preclinical	Very Early	Early	Intermediate	Advanced
	$\bar{X}$ ± SD					
SDMT Correct answers	21.85 ± 18.66	54.50 ± 10.73	43.58 ± 12.88	25.80 ± 9.70	14.75 ± 8.36	1.90 ± 5.56
SDMT Incorrect answers	0.68 ± 1.39	0.37 ± 0.47	0.91 ± 1.08	0.71 ± 1.76	1.03 ± 1.63	0.15 ± 0.67
VF Correct answers	11.55 ± 8.23	24.85 ± 2.41	19.72 ± 5.23	12.50 ± 5.72	10.06 ± 6.45	3.50 ± 4.85
VF Incorrect answers	0.03 ± 0.18	0 ± 0	0 ± 0	0.04 ± 0.21	0.06 ± 0.25	0 ± 0
VF Repetitions	0.68 ± 1.35	0.42 ± 0.78	0.45 ± 1.50	0.86 ± 1.12	0.93 ± 1.81	0.35 ± 0.74
TF I Correct words	6.40 ± 5.68	16.12 ± 4.35	12.25 ± 5.20	6.63 ± 3.53	4.63 ± 3.65	1.40 ± 2.25
TF II Correct words	8.31 ± 8.09	20.75 ± 4.39	13.58 ± 5.10	8.68 ± 4.40	5.50 ± 3.56	14.10 ± 11.66
TF III Correct words	7.05 ± 6.33	18.25 ± 5.77	13.66 ± 4.73	7.63 ± 4.04	5.16 ± 3.53	0.80 ± 1.50
TF I–III Average number of correct words for 3 tests	21.38 ± 18.11	55.37 ± 12.33	39.5 ± 13.20	22.95 ± 10.69	15.56 ± 2.14	3.90 ± 6.87
TF I–III Average number of repetitions for 3 tests	0.61 ± 1.08	0.50 ± 0.84	0.58 ± 0.9	1.00 ± 1.15	0.70 ± 10.52	0.15 ± 0.48
TMT part 1 Filling time	106.76 ± 78.5	27.12 ± 8.37	37.16 ± 9.30	68.31 ± 25.96	122.80 ± 66.12	203.47 ± 68.91
TMT part 1 Correct answers	20.43 ± 9.54	25 ± 0	25 ± 0	25 ± 0	23.03 ± 6.360	6.26 ± 10.84
TMT part 1 Incorrect answers	0.31 ± 0.96	0 ± 0	0 ± 0	0.22 ± 0.75	0.46 ± 1.04	0.52 ± 1.42
TMT part 2 Filling time	169.86 ± 114.43	47.62 ± 10.87	19.72 ± 5.23	149.90 ± 59.84	203.43 ± 56.3	252.36 ± 181.81
TMT part 2 Correct answers	18.89 ± 10.19	25 ± 0	25 ± 0	23.81 ± 4.11	20.90 ± 7.94	3.57 ± 8.55
TMT part 2 Incorrect answers	0.90 ± 1.72	0.12 ± 0.35	0.45 ± 1.50	1.36 ± 1.91	1.40 ± 2.07	0.36 ± 1.38
SCNT Correct answers	36.53 ± 23.96	72.25 ± 13.82	63.25 ± 6.81	44.72 ± 13.87	29.46 ± 14.2	7.80 ± 10.94
SCNT Incorrect answers	0.19 ± 0.59	0 ± 0	0.08 ± 0.28	0.36 ± 0.95	0.20 ± 0.48	0.15 ± 0.48
SCNT Incorrect answers–corrected	0.58 ± 1.02	0.62 ± 0.91	0.58 ± 0.79	0.50 ± 0.96	0.96 ± 1.320	0.10 ± 0.44
SWRT Correct answers	43.03 ± 29.04	89.25 ± 8.24	72.66 ± 23.96	50.22 ± 10.92	36.86 ± 17.60	7.80 ± 13.02
SWRT Incorrect answers	0.05 ± 0.22	0 ± 0	0 ± 0	0.09 ± 0.29	0.06 ± 0.25	0.05 ± 0.22
SWRT Incorrect answers–corrected	0.13 ± 0.47	0 ± 0	0.08 ± 0.28	0.22 ± 0.75	0.13 ± 0.44	0.10 ± 0.30
SIT Correct answers	20.04 ± 15.5	46.25 ± 14.36	34.83 ± 8.24	23.63 ± 6.98	16.03 ± 10.19	2.55 ± 5.28
SIT Incorrect answers	0.52 ± 1.09	0 ± 0	0.58 ± 0.99	0.86 ± 1.24	0.72 ± 1.38	0.05 ± 0.22
SIT Incorrect answers–corrected	0.62 ± 1.02	0.25 ± 0.46	0.75 ± 0.96	1.13 ± 1.48	0.68 ± 0.89	0.05 ± 0.22

$\bar{X}$ ± SD—mean ± standard deviation, SDMT—Symbol Digit Modalities Test, VF—Verbal Fluency test, TF—Total Fluency test, SCNT—Stroop Color-Naming Test, SWRT—Stroop Word Reading test, SIT—Stroop Interference Test, TMT—Trail Making Test.

**Table 4:** Plasma levels of TGF-β1 in the control group and HD patients.

HD	Number of Measurements	TGF-β1 $\bar{X}$ ± SD	TGF-β1 Average Range
Control group	39	648.69 ± 663.35	0–2096.87
All patients	100	611.34 ± 542.79	0–2096.87
Preclinical	12	711.31 ± 556.99	0–1970.48
Very early	12	602.40 ± 634.34	0–2096.87
Early	23	517.93 ± 453.91	0–1647.37
Intermediate	33	543.87 ± 482.81	0–2096.87
Advanced	20	702.60 ± 423.80	94.26–423.80

$\bar{X}$ ± SD—mean ± standard deviation.

**Table 5:** Correlations between plasma levels of TGF-β1 and each parameter of the motor UHDRS determined with Spearman’s test.

Correlations/ HD Stages	All HD		Very Early		Early		Intermediate		Advanced	
	R	*p*	R	*p*	R	*p*	R	*p*	R	*p*
TGF-β1 and tongue protrusion	n.s.	n.s.	n.s.	n.s.	n.s.	n.s.	n.s.	n.s.	0.53	0.01
TGF-β1 and right upper limb tapping	n.s.	n.s.	n.s.	n.s.	n.s.	n.s.	n.s.	n.s.	−0.47	0.03
TGF-β1 and left upper limb tapping	n.s.	n.s.	n.s.	n.s.	0.46	0.02	n.s.	n.s.	−0.47	0.03
TGF-β1 and left upper limb pronation/supination	n.s.	n.s.	n.s.	n.s.	0.47	0.02	n.s.	n.s.	n.s.	n.s.
TGF-β1 and right upper limb pronation/supination	n.s.	n.s.	n.s.	n.s.	0.48	0.01	n.s.	n.s.	n.s.	n.s.
TGF-β1 and torso dystonia	n.s.	n.s.	n.s.	n.s.	n.s.	n.s.	n.s.	n.s.	−0.45	0.04
TGF-β1 and right lower limb dystonia	n.s.	n.s.	n.s.	n.s.	n.s.	n.s.	0.36	0.03	n.s.	n.s.
TGF-β1 and left lower limb dystonia	n.s.	n.s.	n.s.	n.s.	n.s.	n.s.	n.s.	n.s.	−0.44	0.04

R—Spearman’s correlation coefficient, n.s.—not statistically significant.

**Table 6:** Correlations between plasma levels of TGF-β1 and neuropsychological assessment determined with Spearman’s test.

Correlations/ HD Stages	All HD		Preclinical		Very Early		Early		Intermediate		Advanced	
	R	*p*	R	*p*	R	*p*	R	*p*	R	*p*	R	*p*
TGF-β1 and the MMSE test	n.s.	n.s.	n.s.	n.s.	n.s.	n.s.	0.52	0.03	n.s.	n.s.	−0.66	0.01
TGF-β1 and clock drawing test	n.s.	n.s.	n.s.	n.s.	n.s.	n.s.	0.56	0.01	n.s.	n.s.	n.s.	n.s.
TGF-β1 and Beck’s scale	n.s.	n.s.	0.74	<0.01	n.s.	n.s.	n.s.	n.s.	n.s.	n.s.	n.s.	n.s.
TGF-β1 and the independence scale	n.s.	n.s.	n.s.	n.s.	n.s.	n.s.	n.s.	n.s.	0.45	<0.01	n.s.	n.s.

R—Spearman’s correlation coefficient, n.s.—not statistically significant, MMSE—Mini-Mental State Examination.

**Table 7:** Correlations between plasma levels of TGF-β1 and cognitive functions determined with Spearman’s test.

Correlations/ HD Stages	All HD		Preclinical		Very Early		Early		Intermediate		Advanced	
	R	*p*	R	*p*	R	*p*	R	*p*	R	*p*	R	*p*
TGF-β1 and VF Repetitions 0–60 s	−0.25	0.01	n.s.	n.s.	n.s.	n.s.	−0.43	0.04	0.39	0.02	−0.57	<0.01
TGF-β1 and SWRT Correct answers	n.s.	n.s.	n.s.	n.s.	n.s.	n.s.	−0.43	0.04	n.s.	n.s.	n.s.	n.s.
TGF-β1 and SWRT Incorrect answers–corrected	n.s.	n.s.	n.s.	n.s.	n.s.	n.s.	n.s.	n.s.	n.s.	n.s.	−0.46	0.04
TGF-β1 and TMT part 1 Incorrect selections	n.s.	n.s.	n.s.	n.s.	n.s.	n.s.	0.42	0.04	n.s.	n.s.	n.s.	n.s.

R—Spearman’s correlation coefficient, n.s.—not statistically significant, VF—Verbal Fluency test, SWRT—Stroop Word Reading Test, TMT—Trail Making Test.

## Data Availability

Data is contained within the article.
